# Pre-stroke physical activity is associated with post-stroke physical activity and sedentary behavior in the acute phase

**DOI:** 10.1038/s41598-023-48232-z

**Published:** 2023-12-02

**Authors:** Hiroki Tanaka, Gakuto Kitamura, Mayu Tamura, Manabu Nankaku, Masashi Taniguchi, Takayuki Kikuchi, Takakuni Maki, Ryosuke Ikeguchi, Susumu Miyamoto, Ryosuke Takahashi, Shuichi Matsuda, Noriaki Ichihashi

**Affiliations:** 1 Rehabilitation Unit, Kyoto University Hospital, 54 Shogoin-Kawahara-Cho, Sakyo-Ku, Kyoto, 606-8507 Japan; 2 Graduate School of Medicine, Human Health Sciences, Kyoto University, Kyoto, Japan; 3 Department of Neurosurgery, Kyoto University Graduate School of Medicine, Kyoto, Japan; 4 Department of Neurology, Kyoto University Graduate School of Medicine, Kyoto, Japan; 5 Department of Orthopaedic Surgery, Kyoto University Graduate School of Medicine, Kyoto, Japan

**Keywords:** Public health, Quality of life, Therapeutics, Neurological disorders

## Abstract

This study investigated the link between pre-stroke and acute-stage physical activity (PA) and sedentary behavior. Forty individuals with stroke (aged 73.6 ± 8.9 years) were enrolled. Post-stroke activity, including metabolic equivalents (METs), sedentary behavior, light PA, and moderate-to-vigorous PA (MVPA), was measured using a tri-axial accelerometer (ActiGraph wGT3X-BT) over 11 consecutive days starting from the 4th day post-stroke. Pre-stroke PA levels were assessed using the International Physical Activity Questionnaire (IPAQ). We measured skeletal muscle mass index (SMI) and phase angle using a bioelectrical impedance analyzer (Inbody S10) upon admission. Physical therapists assessed the Brunnstrom recovery stage (BRS) within 3 days post-stroke. Total daily activity averaged 1.05 ± 0.05 METs. Throughout the day, 91.2 ± 5.1, 7.6 ± 4.1, and 1.2 ± 1.3% was spent in sedentary behavior, light PA, and MVPA, respectively. Only pre-stroke PA was independently associated with METs (β = 0.66), sedentary behavior (β = −0.58), light PA (β = 0.50), and MVPA (β = 0.71) after adjusting for age, sex, stroke severity, and activities of daily living. This suggests that pre-stroke PA might play a crucial role in reducing sedentary behavior and promoting PA during the acute phase.

## Introduction

Physical activity (PA) is a crucial, modifiable factor that influences not only long-term physical function^1^ but also life expectancy^2^ in survivors of stroke. During the acute phase, increasing PA through early mobilization during hospitalization can contribute to improved functional outcomes post-stroke^3–5^. A previous study using behavior mapping to monitor individuals with stroke every 10 min for 24 h, showed that they spent > 80% of their time either resting in bed or sitting out of bed in the stroke care unit^6^. A recent investigation into 24-h activity in the stroke care unit during the 1st week post-stroke employing accelerometers, demonstrated that the majority of the day (> 90%) was spent engaging in sedentary behavior (energy expenditure of ≤ 1.5 metabolic equivalents [METs])^7^.

To provide more effective rehabilitation for individuals with acute stroke, identifying the individual factors associated with PA during the acute phase is crucial. Although PA in stroke survivors living in the community is generally, lower than that in healthy older individuals^8^, a consensus exists that increasing PA after stroke offers health benefits and reduces stroke recurrence^9,10^. A recent review reported that PA was strongly associated with physical function in community-dwelling stroke survivors^11^. Therefore, hypothesizing that motor function with paralysis and muscle composition might affect PA during the acute post-stroke period. However, it remains unclear which individual patient factors are associated with PA in the acute phase, where medical issues often hinder PA and its compensation relies on medical staff.

High levels of pre-stroke PA are linked to a reduced stroke severity and improved of long-term functional outcomes^12–15^. Although stroke severity is a factor directly related to functional outcomes, higher pre-stroke PA is associated with better long-term outcomes, even when corrected for stroke severity at onset^15^. In other words, PA before stroke includes some factors that improve the outcomes in addition to reducing stroke severity. Higher PA in the acute phase after a stroke leads to enhanced functional outcomes^3–5^. If pre-stroke PA promotes post-stroke PA, the relationship between pre-stroke PA and long-term functional outcomes could be clarified more accurately. However, no study has examined the relationship between pre- and post-stroke PA during the acute phase.

This study aimed to investigate the relationships between post-stroke PA and sedentary behavior in the acute phase and patient characteristics such as pre-stroke PA, physical function at stroke onset, and musculoskeletal status on admission. We hypothesized that higher pre-stroke PA would be related to more time engaged in PA and less sedentary time in acute phase of stroke. Furthermore, we also anticipated that better physical function and musculoskeletal status would correlate with high-intensity PA.

## Results

### Participant characteristics

Among all patients hospitalized for stroke during the study period, 46 participants meeting the inclusion criteria were enrolled in this study. Six participants were excluded due to insufficient accelerometer measurement (< 7 days) resulting from early discharge or transfer to another convalescent rehabilitation hospital within the 1st week. Therefore, 40 participants were enrolled in the present study. Participants’ characteristics are shown in Table 1.

**Table 1:** Characteristics of the participants.

Sex	Male, *n* = 27 Female, *n* = 13
Age, years	73.6 ± 8.9
Height, cm	161.4 ± 9.2
Weight, kg	63.7 ± 12.7
Diagnosis	CI, *n* = 34 ICH, *n* = 6
Side of paresis	Right, *n* = 23 Left, *n* = 17
NIHSS	4.2 ± 3.9
BRS, median (IQR)	5 (3.75, 6)
FIM motor score	25.0 ± 13.7
FIM cognitive score	29.2 ± 7.31
Comorbidities, n (%)	
- Previous stroke	5 (12.5)
- Hypertension	29 (72.5)
- Atrial fibrillation	3 (7.5)
- Dyslipidemia	16 (40)
- Diabetes mellitus	17 (42.5)
- Chronic kidney disease	1 (2.5)
Smoking, n (%)	21 (52.5)
IPAQ	
- MET-min/week	1636.6 ± 1086.4
Accelerometer	
- METs/day	1.1 ± 0.1
- % in Sedentary	91.2 ± 5.1
- % in Light PA	7.6 ± 4.1
- % in MVPA	1.2 ± 1.3

CI: Cerebral infarction; ICH: Intracerebral hemorrhage; NIHSS: National Institutes of Health stroke scale; BRS: Brunnstrom recovery stage; FIM: Functional independence measure; IPAQ: International Physical Activity Questionnaire; METs: Metabolic equivalents; PA: Physical activity; MVPA: Moderate to vigorous physical activity.

### Characteristics of post-stroke activity

As an indicator of compliance with post-stroke activity measurements, participants averaged 10.0 ± 1.4 days with the accelerometer within the 11-day period, amounting to an average of 1269.5 ± 148.9 min/day out of the total 1440 min. These findings suggested a high compliance rate with the 24-h accelerometer measurements.

**Figure 1:**
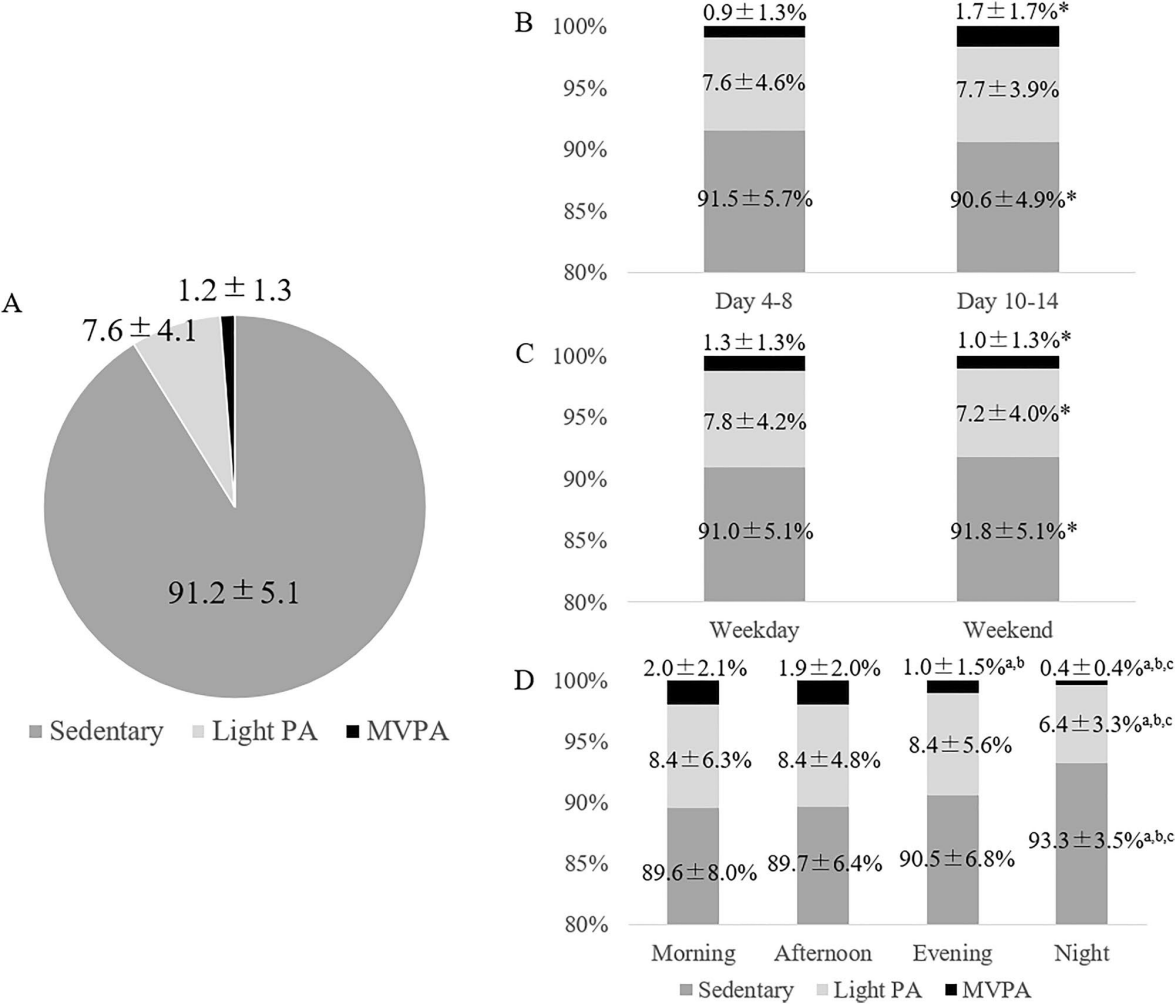
Sedentary behavior and physical activity. (**A**) Average percentage of sedentary behavior and physical activity for 11 days. Comparisons include: (**B**) between the first 5 days (4th to 8th day from stroke onset) and the subsequent 5 days (10th to 14th day); (**C**) between weekdays and weekends; (**D**) between morning (7:00–12:00), afternoon (12:00–17:00), evening (17:00–22:00), and night (22:00–7:00). *Denotes a significant difference between day 4–8 and day 10–14 or between weekdays and weekends (*p* < 0.05). The letters “a”, “b”, and “c” represent significant difference from morning, afternoon, and evening, respectively (*p* < 0.05). PA: Physical activity, MVPA: Moderate to vigorous physical activity.

The average total activity amounted to 1.05 ± 0.05 METs/day. The mean percentage and variability of each activity intensity per day is shown in Fig. 1. Sedentary behavior was significantly longer during the first 5 days compared with the subsequent 5 days (91.5 ± 5.7% vs. 90.6 ± 4.9%, *p* = 0.01); on weekends compared with weekdays (91.8 ± 5.1% vs. 91.0 ± 5.1%, *p* < 0.01); and during the night compared with the morning, afternoon, and evening (93.3 ± 3.5% vs. 89.6 ± 8.0%, 89.7 ± 6.4%, and 90.5 ± 6.8%, *p* < 0.01, *p* < 0.01, and *p* < 0.01). Light PA was significantly lower on weekends compared with weekdays (7.2 ± 4.0% vs. 7.8 ± 4.2%, *p* < 0.01); and in the night compared with the morning, afternoon, and evening (6.4 ± 3.3% vs. 8.4 ± 6.3%, 8.4 ± 4.8%, and 8.4 ± 5.6%, *p* = 0.01, *p* < 0.01, and *p* < 0.01). Moderate-to-vigorous PA (MVPA) was significantly lower on the first 5 days compared with the subsequent 5 days (0.9 ± 1.3% vs. 1.7 ± 1.7%, *p* < 0.01); on weekends compared with weekdays (1.0 ± 1.3% vs. 1.3 ± 1.3%, *p* = 0.02); and during the night compared with the morning, afternoon, and evening (0.4 ± 0.4% vs. 2.0 ± 2.1%, 1.9 ± 2.0%, and 1.0 ± 1.5%, *p* < 0.01, *p* < 0.01, and *p* < 0.01).

### Multiple regression analysis for sedentary behavior and PA

**Figure 2:**
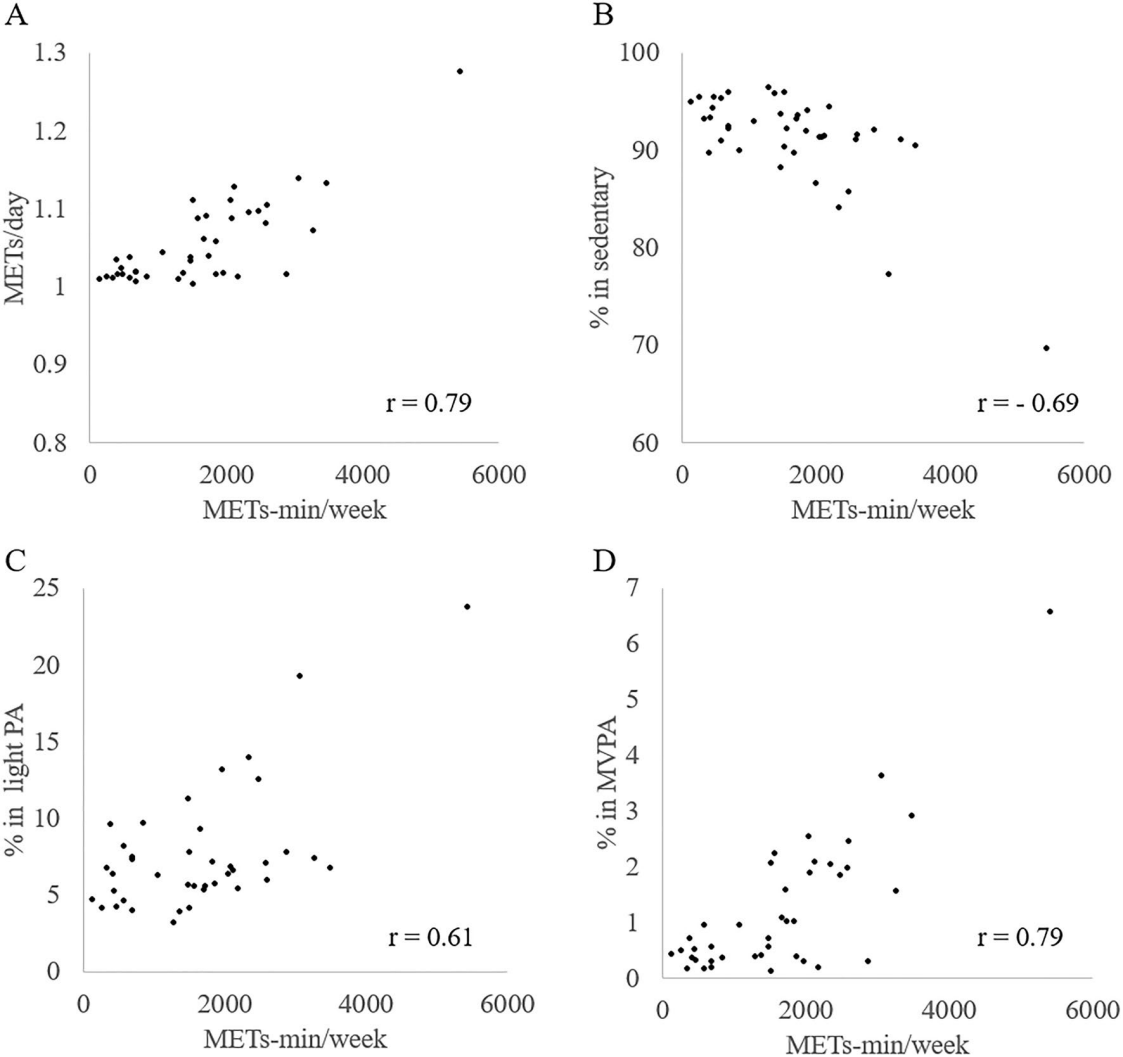
The relationships of physical activity between before and after stroke. The scatterplots illustrate the correlations between METs-min/week calculated from IPAQ and (**A**) total activity (METs/day); (**B**) % in sedentary; (**C**) % in light PA; (**D**) % in MVPA. METs: Metabolic equivalents, IPAQ: International Physical Activity Questionnaire, PA: Physical activity, MVPA: Moderate to vigorous physical activity.

Table 2 presents the results of the multiple linear regression analysis. Pre-stroke PA assessed by the International Physical Activity Questionnaire (IPAQ), skeletal muscle mass index (SMI), and Brunnstrom recovery stage (BRS) independently correlated with METs/day (model 1). After adjusting for age, sex, the National Institutes of Health Stroke Scale (NIHSS), and the Functional Independence Measure (FIM) motor score, a higher pre-stroke PA time per week was associated with the daily average of METs after the stroke (model 2). Only pre-stroke PA was independently associated with sedentary behavior and light PA (model 1). Pre-stroke PA and BRS were independently associated with MVPA (model 1). After adjustment, higher pre-stroke PA was associated with shorter sedentary behavior, longer light PA, and greater MVPA time (model 2). Scatterplots illustrating the relationship between pre- and post-stroke PA are displayed in Fig. 2.

**Table 2:** Multiple regression analysis for sedentary behavior and physical activity.

	METs/day		Sedentary		Light PA		MVPA	
	β	*P*	β	*P*	β	*P*	β	*P*
Model 1								
IPAQ	0.62	< 0.01	−0.62	< 0.01	0.56	< 0.01	0.68	< 0.01
SMI	0.27	0.02	−0.20	0.17	0.19	0.24	0.18	0.12
Phase angle	0.03	0.78	−0.07	0.62	0.09	0.57	0.00	0.97
BRS	0.30	< 0.01	0.04	0.78	−0.12	0.40	0.25	0.02
R^2^	0.72		0.50		0.41		0.68	
Model 2								
Adjusted for age, sex, NIHSS, FIM motor score								
IPAQ	0.66	< 0.01	−0.58	< 0.01	0.50	< 0.01	0.71	< 0.01
SMI	0.26	0.08	−0.25	0.19	0.25	0.23	0.20	0.20
Phase angle	0.11	0.38	−0.03	0.84	0.03	0.88	0.04	0.74
BRS	0.19	0.18	0.13	0.51	−0.21	0.31	0.18	0.25
Age	0.15	0.23	0.13	0.44	−0.20	0.26	0.14	0.30
Sex	0.07	0.52	0.04	0.75	−0.07	0.66	0.04	0.71
NIHSS	0.03	0.81	0.30	0.12	−0.37	0.07	0.01	0.94
FIM motor score	0.12	0.31	0.07	0.65	−0.11	0.54	0.05	0.70
R^2^	0.71		0.49		0.41		0.66	

METs: Metabolic equivalents; PA: Physical activity; MVPA: Moderate to vigorous physical activity; IPAQ: International Physical Activity Questionnaire; SMI: Skeletal Muscle mass Index; BRS: Brunnstrom recovery stage; NIHSS: National Institutes of Health Stroke Scale; FIM: Functional independence measure.

## Discussion

In our study, we used a tri-axial accelerometer to measure total activity (METs), sedentary behavior, light PA, and MVPA in individuals with acute stroke. Consistent with our hypothesis, a higher level of pre-stroke PA was associated with spending longer time in post-stroke PA and shorter time in sedentary behavior. The expected relationship between physical function, musculoskeletal status, and high-intensity PA was supported by the significant correlation between BRS or SMI and METs or MVPA. However, these relationships lost significance after adjusting for moderator variables.

The most noteworthy finding of our study was the extensive association between pre-stroke PA and post-stroke activity. To the best of our knowledge, this report is the first to established that PA before stroke is related to sedentary behavior and PA in the acute post-stroke period. Low PA before stroke may pose a risk for prolonged sedentary behavior and reduced PA during the acute phase, potentially influencing long-term outcomes as shown in a previous study^15^.

Our results showed that individuals with acute stroke spent > 90% of their time in sedentary behavior, approximately 8% in light PA, and approximately 1% in MVPA. This result was comparable with previous studies that reported sedentary behavior and PA in the acute stroke phase^7,16^. Another study demonstrated that PA in individuals with ischemic stroke was 71% lower than that in individuals with transient ischemic attack as a control group^17^. In this previous study^17^, it was unclear why PA was lower in individuals with stroke than in those with transient ischemic attacks, despite involving the same hospitalization conditions. Therefore, investigating the characteristics of PA in individuals with stroke, including time of day utilized and diurnal variations, is crucial. Additionally, we identified that sedentary behavior was more prolonged in the early post-onset period, on weekends, and during the evening. These results suggest that, aside from environmental factors, such as caregiver availability and rehabilitation resources may contribute to lower PA in patients with stroke. Therefore, interventions targeting individuals with extended sedentary behavior due to these environmental factors should be considered.

Although total activity (METs/day) and MVPA correlated with the motor function of the affected lower limb (BRS), sedentary behavior and light PA were not associated with BRS. This indicates that high physical motor functioning is necessary for engaging in higher-intensity PA beyond walking, but it is not a prerequisite for reducing sedentary behavior. Light activities, such as self-care tasks (eating, dressing, transferring, and toileting), fall within the 1.5–2.5 METs range^18^. Hence, even if performing high-intensity PAs such as walking or carrying loads is challenging, increasing the frequency and time spent on these self-care activities can reduce sitting time. Exercise has been known to mitigate fatigue and depression^19,20^, and pre-stroke PA habits can provide protection against depression^21^. Thus, PA seems to be influenced by both physical function and psychological states, including motivation, fatigue, and depression. Consequently, stable psychological conditions due to pre-stroke PA habits may encourage an increase in light PA, while simultaneously associating pre-stroke PA with sedentary behavior.

Adequate compliance with an accelerometer measure was obtained with a wearing period of 10.0 ± 1.4 days for 11 days and a wearing time of 1269.5 ± 148.9 min for 1440 min. Both the wearing period and time in our study were longer than those in previous studies of the acute stroke phase^7,16,17^. This strength of our study would increase the validity of our results, as activity can vary over different times and days. However, limitations exist in the present study. Wearing the accelerometer on the lower back or thigh is recommended to accurately reflect body movement^22^. In this study, it was placed on the ankle of the non-paretic lower extremity for wearing compliance and to prevent skin damage. Furthermore, the type of movement causing a measured intensity of activity was unknown because we had no records of behavior for PA measurement in this study. Therefore, one must consider the validity of the results concerning the specific movements involved, and exercising caution when comparing these results with those from previous studies is crucial. Furthermore, we included only those who understood and consented to the study immediately after the onset of stroke. Therefore, selection bias would exist because those who were relatively well-functioning in the overall population of individuals with stroke were recruited. However, the possibility of recall bias or the underestimation of IPAQ cannot be ruled out. Although the association between PA before and after stroke was found in controlling for potentially confounding variables, an outlier may have influenced the results as Fig. 2 shows. Moreover, the number of cases that could be recruited is small, and care must be taken in interpreting the statistical results. In the future, investigating interventions aimed at increasing PA in severe cases may be crucial. Finally, other factors^11^, such as depression, fatigue, self-efficacy, and quality of life, related to post-stroke PA were not included in this study. Furthermore, we excluded patients with unstable medical conditions including those at high risk of stroke recurrence, therefore, almost all participants were allowed to leave the bed freely, without any bed rest restrictions instructed by their doctors. However, we had not considered the possibility of recurrence and the risk of falls although these factors could have influenced reduced PA because participants were unable to increase their PA owing to these medical conditions.

The results of our study revealed that individuals with acute stroke devote > 90% of their time to sedentary behavior (with an energy expenditure of ≤ 1.5 METs) and allocate less time to engaging in PA. Moreover, pre-stroke PA was strongly associated with post-stroke activity (METs, sedentary behavior, light PA, and MVPA). This implies that low pre-stroke PA may pose a risk for reduced PA during the acute phase of stroke.

## Methods

### Study design and participants

A single center observational study was conducted. Inclusion criteria: Patients hospitalized in Kyoto University Hospital; aged 20–90 years old; diagnosed with stroke (cerebral infarction or hemorrhage) by board-certified neurologists or neurosurgeons at Kyoto University Hospital from January 2021 to June 2022, and who provided consent within 3 days of stroke onset. Patients who could not agree with the study concept, follow instructions, or sign the consent form due to a significant decline in consciousness level, cognitive or motor functioning, as well as those who had experienced limitations of physical movement before admission, were unable to participate owing to their medical conditions including those at high risk of stroke recurrence, or were discharged or transferred to another hospital without 7 days of accelerometer measurement, were excluded. All participants were fully informed of the procedures and purpose of the study, which conformed to the tenets of the Declaration of Helsinki. Written informed consent was obtained from all participants. This study was approved by the ethics committee of Kyoto University Graduate School and the Faculty of Medicine (R2748).

### PA

Pre-stroke PA was investigated using the IPAQ Short Form, which was validated in the investigation of PA^23^. The duration of time per day and frequency per week were investigated for each of the four intensity levels: (1) vigorous-intensity activities such as carrying heavy loads and jogging, (2) moderate intensity activities such as carrying light loads, (3) walking, and (4) sitting for the usual 7-day recall. The total value of each activity intensity multiplied by the time in a week (METs-min/week) was calculated from the results obtained according to the IPAQ guidelines^24^.

Post-stroke activity was measured using a tri-axial accelerometer (ActiGraph wGT3X-BT, ActiGraph LLC, Pensacola, FL, USA) placed on the ankle of the non-paralyzed side. The accelerometer was worn daily for 24 h, except during bathing or examinations from the day of consent to discharge from the hospital. The data for 11 consecutive days (from the 4^th^ day following stroke onset) were used in the analysis to align the measurement period for each participant. The analysis of the accelerometer was conducted using specialized software (ActiLife Version 6.13.4; ActiGraph LLC, Pensacola, FL, USA). The total activity (METs) and the total time of each activity intensity (sedentary behavior, light PA, MVPA) were calculated from the acceleration data using the aforementioned software. The cut-off points for activity intensity were based on the validity study^25^: sedentary behavior,  ≤ 99 counts per min; light PA, 100–1951 counts per min; MVPA,  ≥ 1952 counts per min.

### Outcome measures

NIHSS score (a 15-item impairment scale, with a score range of 0–42, encompassing no neurological deficit to very severe neurological deficits)^26^, BRS (a measure of motor function recovery assessing spasticity and involuntary muscle movement, ranging from stage I–VI)^27^, and the FIM motor score^28^ were measured by the doctor or physical therapist as an index of disease severity and affected lower limb motor function. NIHSS was evaluated by the doctor right after admission or immediately following thrombectomy or recombinant tissue plasminogen activator therapy. BRS of the paralyzed lower extremity and the FIM motor score was evaluated by a physical therapist within 3 days after stroke onset. SMI and phase angle were measured by using a bioelectrical impedance analyzer (InBody S10; InBody Co., Ltd., Seoul, Korea) on the same day as the BRS and FIM measurements. After resting, the participants were measured for body composition in the supine position. Please refer to previous literature for detailed measurement procedures^29^. SMI and phase angle were used to evaluate body composition in terms of both muscle quantity and quality^30,31^. SMI was calculated by dividing appendicular skeletal muscle mass by height squared. The phase angle at 50 kHz was calculated using the following equation: phase angle (°) = arctan (reactance/resistance) × (180/π).

### Statistical analysis

Statistical analyses were performed using SPSS version 28.0 (IBM, Armonk, NY, USA). The percentage of sedentary behavior, light PA, and MVPA for 11 days were calculated. We compared each activity during the first 5 days and the last 5 days, both on weekdays and weekends, as well as different time segments: morning (7:00–12:00), afternoon (12:00–17:00), evening (17:00–22:00), and night (22:00–7:00). We employed a paired t-test with Shaffer correction for this analysis. In the analysis of the initial and subsequent halves of the measurement, the analysis interval was divided into two equal parts: the first 5 days (4th–8th) and the subsequent 5 days (10th–14th day). Therefore, data for PA on the 9th day were not included in this analysis.

Multiple linear regression analysis was performed to determine predictors for each activity (METs, sedentary behavior, light PA, MVPA). Pre-stroke PA (IPAQ), SMI, phase angle, and BRS were set as an independent variable (model 1). Subsequently, age, sex, NIHSS, and FIM motor score were set as moderator variable (model 2). A *p*-value of < 0.05 was considered statistically significant.

## Data Availability

The datasets used and/or analyzed during the current study are available from the corresponding author on reasonable request.

## References

[1] Boysen G, Krarup LH (2009). Benefits of physical activity for stroke survivors. Expert. Rev. Neurother..

[2] Loprinzi PD, Addoh O (2018). Accelerometer-determined physical activity and all-cause mortality in a national prospective cohort study of adults post-acute stroke. Am. J. Health Promot..

[3] Hokstad A (2016). Upright activity within the first week after stroke is associated with better functional outcome and health-related quality of life: A Norwegian multi-site study. J. Rehabilit. Med..

[4] Askim T, Bernhardt J, Salvesen O, Indredavik B (2014). Physical activity early after stroke and its association to functional outcome 3 months later. J. Stroke Cerebrovasc. Dis..

[5] Bernhardt J (2016). Prespecified dose-response analysis for a very early rehabilitation trial (AVERT). Neurology.

[6] Bernhardt J, Dewey H, Thrift A, Donnan G (2004). Inactive and alone: physical activity within the first 14 days of acute stroke unit care. Stroke.

[7] Mattlage AE (2015). Use of Accelerometers to Examine Sedentary Time on an Acute Stroke Unit. J. Neurol. Phys. Ther.: JNPT.

[8] English C, Manns PJ, Tucak C, Bernhardt J (2014). Physical activity and sedentary behaviors in people with stroke living in the community: a systematic review. Phys. Ther..

[9] Warburton DER, Nicol CW, Bredin SSD (2006). Health benefits of physical activity: the evidence. Can. Med. Assoc. J..

[10] Howard VJ, McDonnell MN (2015). Physical activity in primary stroke prevention: just do it!. Stroke.

[11] Thilarajah S (2018). Factors associated with post-stroke physical activity: a systematic review and meta-analysis. Arch. Phys. Med. Rehabil..

[12] Reinholdsson M, Palstam A, Sunnerhagen KS (2018). Prestroke physical activity could influence acute stroke severity (part of PAPSIGOT). Neurology.

[13] Spartano NL, Bernhardt J (2018). Prestroke physical activity to reduce stroke severity: Moving to lower risk with light activity. Neurology.

[14] Hung SH (2021). Pre-stroke physical activity and admission stroke severity: A systematic review. Int. J. Stroke.

[15] Krarup LH (2008). Prestroke physical activity is associated with severity and long-term outcome from first-ever stroke. Neurology.

[16] Nozoe M (2016). Physical activity in acute ischemic stroke patients during hospitalization. Int. J. Cardiol..

[17] Strømmen AM, Christensen T, Jensen K (2014). Quantitative measurement of physical activity in acute ischemic stroke and transient ischemic attack. Stroke.

[18] Ainsworth BE (1993). Compendium of physical activities: classification of energy costs of human physical activities. Med. Sci. Sports Exerc..

[19] Graven C, Brock K, Hill K, Joubert L (2011). Are rehabilitation and/or care co-ordination interventions delivered in the community effective in reducing depression, facilitating participation and improving quality of life after stroke?. Disabil. Rehabilit..

[20] Zedlitz AM, Rietveld TC, Geurts AC, Fasotti L (2012). Cognitive and graded activity training can alleviate persistent fatigue after stroke: a randomized, controlled trial. Stroke.

[21] Bovim MR (2019). Relationship between pre-stroke physical activity and symptoms of post-stroke anxiety and depression: An observational study. J. Rehabilit. Med..

[22] Arvidsson D, Fridolfsson J, Börjesson M (2019). Measurement of physical activity in clinical practice using accelerometers. J. Intern. Med..

[23] Craig CL (2003). International physical activity questionnaire: 12-country reliability and validity. Med Sci Sports Exerc.

[24] *Guidelines for the data processing and analysis of the "International Physical Activity Questionnaire"*. (The IPAQ Group, 2005).

[25] Freedson PS, Melanson E, Sirard J (1998). Calibration of the computer science and applications. Inc. Accelerometer. Med. Sci. Sports Exerc..

[26] Brott T (1989). Measurements of acute cerebral infarction: a clinical examination scale. Stroke.

[27] Brunnstrom S (1966). Motor testing procedures in hemiplegia: based on sequential recovery stages. Phys. Ther..

[28] Kidd D (1995). The Functional Independence Measure: a comparative validity and reliability study. Disabil. Rehabilit..

[29] Tanaka H (2022). Association of physical activity and nutritional intake with muscle quantity and quality changes in acute stroke patients. J. Stroke Cerebrovasc. Dis..

[30] Bourgeois B (2019). Improved strength prediction combining clinically available measures of skeletal muscle mass and quality. J Cachexia Sarcopenia Muscle.

[31] Tomeleri CM (2019). Phase angle is moderately associated with muscle quality and functional capacity, independent of age and body composition in older women. J. Geriatr. Phys. Ther..

